# Exercise Training and Calorie Restriction Influence the Metabolic Parameters in Ovariectomized Female Rats

**DOI:** 10.1155/2015/787063

**Published:** 2015-03-19

**Authors:** Anikó Pósa, Renáta Szabó, Krisztina Kupai, Anett Csonka, Zita Szalai, Médea Veszelka, Szilvia Török, Lejla Daruka, Csaba Varga

**Affiliations:** Department of Physiology, Anatomy and Neuroscience, University of Szeged, Kozep Fasor 52, Szeged 6726, Hungary

## Abstract

The estrogen deficiency after menopause leads to overweight or obesity, and physical exercise is one of the important modulators of this body weight gain. Female Wistar rats underwent ovariectomy surgery (OVX) or sham operation (SO). OVX and SO groups were randomized into new groups based on the voluntary physical activity (with or without running) and the type of diet for 12 weeks. Rats were fed standard chow (CTRL), high triglyceride diet (HT), or restricted diet (CR). The metabolic syndrome was assessed by measuring the body weight gain, the glucose sensitivity, and the levels of insulin, triglyceride, leptin, and aspartate aminotransferase transaminase (AST) and alanine aminotransferase (ALT). The exercise training combined with the CR resulted in improvements in the glucose tolerance and the insulin sensitivity. Plasma TG, AST, and ALT levels were significantly higher in OVX rats fed with HT but these high values were suppressed by exercise and CR. Compared to SO animals, estrogen deprivation with HT caused a significant increase in leptin level. Our data provide evidence that CR combined with voluntary physical exercise can be a very effective strategy to prevent the development of a metabolic syndrome induced by high calorie diet.

## 1. Introduction

The metabolic syndrome (MS), usually caused by high calorie diet and a lack of physical activity, covers a heterogeneous cluster of obesity-related diseases. This syndrome is common and its prevalence increases with the menopause and in an estrogen-deficiency state [1]. With the recent dramatic increase in life expectancy, many women are now spending a large part of their lives in a postmenopausal state, and an investigation of strategies to prevent or attenuate the deleterious effects associated with the ovarian hormone decrease is therefore necessary [2].

Ovariectomized (OVX) animals have been used as experimental models of obesity from a limited estrogen function. Estrogen depletion is associated with an increased visceral adipose tissue mass. The increased fat mass may be explained by changes in energy expenditure, because the menopausal transition leads to decreased energy expenditure and reduced fat oxidation [3]. Female aromatase knockout mice, which are unable to synthesize estrogen, display increased body weight and adipocyte hypertrophy, demonstrating the impact of an estrogen deficiency on fat accumulation [4].

Hormone replacement therapy (HRT) has been used to treat estrogen deficiency symptoms. However, there has subsequently been debate which concerns the negative overall risk-benefit ratio of HRT and alternative strategies for the treatment of menopausal and postmenopausal disorders [5]. Exercise training in animals decreases fat deposition, enhances insulin sensitivity, improves the glucose-stimulated insulin response, and increases the glucose transporter concentration [6]. However, voluntary physical exercise training combined with a normal or calorie-restricted or a high-fat phytoestrogen-free nutrition in OVX rats has not been investigated.

We set out to investigate the effects of exercise training combined with dietary restriction as a nonpharmacological intervention with the aim of controlling the effect of estrogen depletion, including obesity, hypertension, glucose tolerance, dyslipidemia, and systemic inflammation. The impact of OVX-induced obesity on metabolic profiles is a matter of current interest. Thus, in the present study we tested whether 12 weeks of exercise training and/or calorie restriction (CR) would improve the metabolic parameters in OVX rats as compared with normal and high-fat diet-fed OVX rats, mimicking the food habits of humans living in western countries.

## 2. Methods

### 2.1. Animals and Experimental Design

This study was performed in accordance with the European Community guidelines on the care and use of laboratory animals and had been approved by the local Institutional Ethics Committee at the University of Szeged.

At 10 wk of age, female Wistar rats (Toxi-Coop Zrt., Hungary) were anesthetized and underwent ovariectomy surgery (OVX) or sham operation (SO). The OVX procedure was performed via a ventral abdominal midline small incision through which the ovaries were bilaterally clamped and removed. The uterine horns were tied and the uterus was left intact. After surgery, the animals were maintained under good conditions to allow them to recover. In the SO procedure, the ovaries were exteriorized to create similar stress, but were not clamped and removed. After a 4-week resting period to verify the OVX-induced menopause, the serum estrogen levels were checked via estrogen quantitative enzyme-linked immunosorbent assay (ELISA) according to the manufacturer's directions (Quantikine rat Estrogen Elisa kit, R&D Systems Inc.). After the resting period, the OVX and SO groups were each subdivided into two new groups, based on the type of diet and recreational exercise. During a 12-week period (with or without training) the rats were fed standard chow (CTRL) subgroup, a high-triglyceride diet (HT) subgroup (60% standard chow with 40% fat), or 50% restricted food diet (CR, 50% of the daily standard chow consumption) subgroup. The daily food consumption was determined as the difference between the amount of chow supplied and the amount of chow remaining.

**Figure 1:**
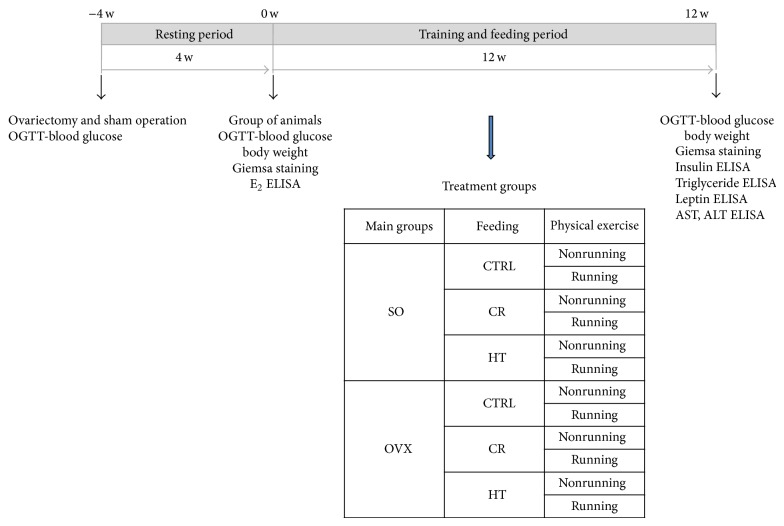
Experimental design of the study. SO = sham-operated, OVX = ovariectomized, CTRL = standard chow, CR = calorie restriction, HT = high-triglyceride, w = weeks, E_2 _= estrogen, OGTT = oral glucose tolerance test, AST = aspartate aminotransferase transaminase, and ALT = alanine aminotransferase.

The running animals (R subgroup) were placed individually into cages fitted with a running-wheel and were allowed free access to the wheel for 24 h per day for 12 weeks. The exercising protocol, defined as a voluntary wheel-running model, was selected in an effort to isolate the effects of exercising from the additional stress associated with forced exercise protocols. Control (nonrunning) rats were placed in standard holding cages without a running-wheel for the same period. The experimental design of the study is presented in Figure 1.

Each rat was labeled and weighed weekly during the experimental period.  Table 1 shows the changes in body weight during the 12-week training and feeding period.

### 2.2. Oral Glucose Tolerance Test (OGTT)

The blood glucose level was measured by means of the OGTT at the week-4, 0 and the end of the 12-week treatment period. The serum levels of insulin were measured by means of the OGTT at the end of the 12-week treatment period. After a 12 h fasting period tail blood was taken before the application of glucose by oral gavage (0.1 g/kg bw) and 30, 60, and 120 min afterwards. Blood samples were collected to determine insulin levels (measured by ELISA, Sunred Biological Technology Co., Shanghai). The blood glucose was analyzed via Accu Check Active strips. Only the 12-week data are presented.

### 2.3. Determination of Insulin, Triglyceride, Leptin, Aspartate Aminotransferase Transaminase (AST), and Alanine Aminotransferase (ALT)

Blood samples were collected at the end of the 12-week treatment period. All samples were centrifuged and the serum was stored at −20°C until analysis. Serum levels of insulin and plasma levels of triglyceride, leptin, AST, and ALT were determined by ELISA according to the manufacturer's instructions (Sunred Biological Technology Co., Shanghai).

### 2.4. Statistical Analysis

The results reported in the table and figures are expressed as means ± S.E.M. Differences between groups were determined with Student's* t*-test and One Way ANOVA Analysis with Shapiro-Wilk normality test and Holm-Sidak post hoc method. *P* values less than 0.05 were considered significant.

## 3. Results

### 3.1. Body Weight

Table 1 lists the changes in body weight before and after the 12-week treatment period. As expected, at the start of the training and feeding period the OVX rats exhibited the highest body weight. After the 12-week treatment period, the weight of the OVX which was found to have rats further increased, whereas the CR alone (14%, *P* < 0.05) or in combination with physical exercise (28%, *P* < 0.05) resulted in a weight reduction.

### 3.2. Glucose Levels and Glucose Tolerance

**Figure 2:**
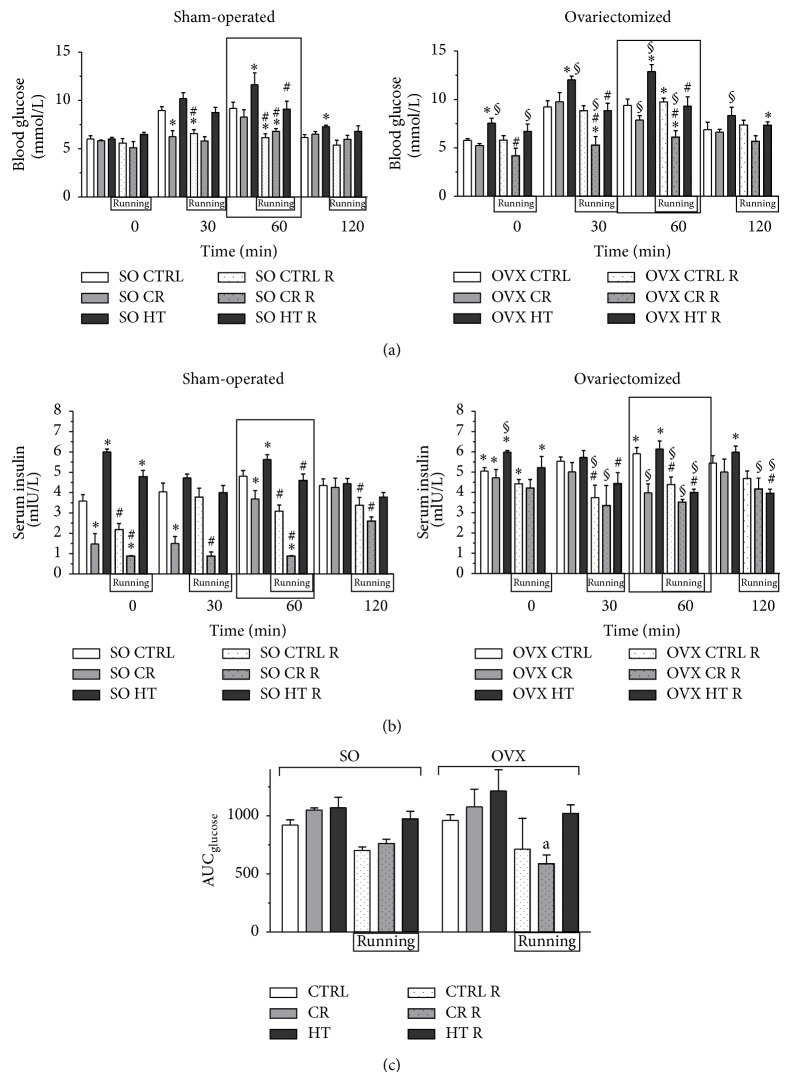
(a): Effects of 12-week wheel-running exercise and nutrition on the level of blood glucose (expressed in mmol/L) before (0 min) and after the oral glucose tolerance test (OGTT). Data are shown as means ± S.E.M. *n* = 10–12. Statistical significance: ^*^ *P* < 0.05 relative to the SO CTRL group at 0, 30, 60, and 120 min, and ^#^ *P* < 0.05 is a significant difference between the running (R) and nonrunning groups at 0, 30, 60, and 120 min, ^§^ *P* < 0.05 relative to the OVX CTRL group at 0, 30, 60, and 120 min. SO = sham-operated, OVX = ovariectomized, CTRL = standard chow, CR = calorie restriction, HT = high-triglyceride. The most significance differences are highlighted in box. (b): Effects of 12-week wheel-running exercise and nutrition on the serum levels of insulin (expressed in mIU/L) before (0 min) and after the oral glucose tolerance test (OGTT). Means ± S.E.M. *n* = 10. Statistical significance: ^*^ *P* < 0.05 relative to the SO CTRL group at 0, 30, 60, and 120 min, and ^#^ *P* < 0.05 is a significant difference between the running (R) and nonrunning groups at 0, 30, 60, and 120 min, ^§^ *P* < 0.05 relative to the OVX CTRL group at 0, 30, 60, and 120 min. The most significance differences are highlighted in box. SO = sham-operated, OVX = ovariectomized, CTRL = standard chow, CR = calorie restriction, HT = high-triglyceride. (c): The effects of 12-week wheel-running exercise and nutrition on the areas under the curve (AUC) for glucose after the OGTT. Data are shown as means ± S.E.M. *n* = 5. Statistical significance: ^a^ *P* < 0.05 relative to the OVX HT group. SO = sham-operated, OVX = ovariectomized, CTRL = standard chow, CR = calorie restriction, HT = high-triglyceride, and AUC = area under curve.

Changes in glucose sensitivity, induced by OGTT after the 12-week treatment period, are presented at each time (0, 30, 60, and 120 min) in Figure 2(a). And the area under the curve for glucose in Figure 2(c) was determined. In both the SO and the OVX rats, wheel-running exercise resulted in an improvement in glucose tolerance. The data clearly demonstrate that voluntary exercise training associated with a CRled with the most effective improvement of the glucose sensitivity (in the SO rats at 60 min 28%, *P* < 0.05; in the OVX rats at 60 min 35%, *P* < 0.05).

### 3.3. Serum Insulin Levels

Serum insulin measurements at the end of the 12-week treatment period revealed an augmented insulin level in the OVX rats as compared with the SO CTRL subgroup. The HT diet alone significantly increased the insulin level in the SO group (67%, *P* < 0.05). The CR improved the insulin level in both the OVX and SO groups (59%, *P* < 0.05). The strongest reductions of the insulin level were observed in the SO and the OVX animals which participated in the CR and running. However, the 12 weeks of exercise caused a more significant reduction in 60 min (in the SO CR R rats 76%, *P* < 0.05; in the OVX CR R rats 40%, *P* < 0.05). Data are presented in Figure 2(b).

### 3.4. Plasma Triglyceride (TG) Levels

**Figure 3:**
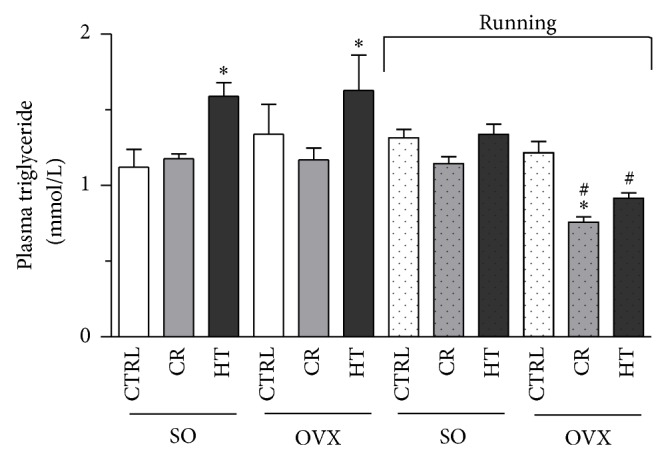
Effects of 12-week wheel-running exercise and nutrition on the plasma levels of triglyceride (expressed in mmol/L). Results are shown as means ± S.E.M. *n* = 12. Statistical significance: ^*^ *P* < 0.05 relative to the SO CTRL group, and ^#^ *P* < 0.05 is the significant difference between the running (R) and nonrunning groups. SO = sham-operated, OVX = ovariectomized, CTRL = standard chow, CR = calorie restriction, and HT = high-triglyceride.

Figure 3 shows the plasma levels of TG measured by ELISA. The highest TG levels were observed in both the SO (42%, *P* < 0.05) and the OVX rats (45%, *P* < 0.05) fed with the HT diet and were significantly higher as compared with the SO CTRL subgroup. However, the combined effects of the 12-week exercise and the CR decreasing the TG levels only in the OVX animals (33%, *P* < 0.05) and the exercise training alone were also effective in the OVX HT subgroup (44%, *P* < 0.05).

### 3.5. Plasma Leptin Concentrations

**Figure 4:**
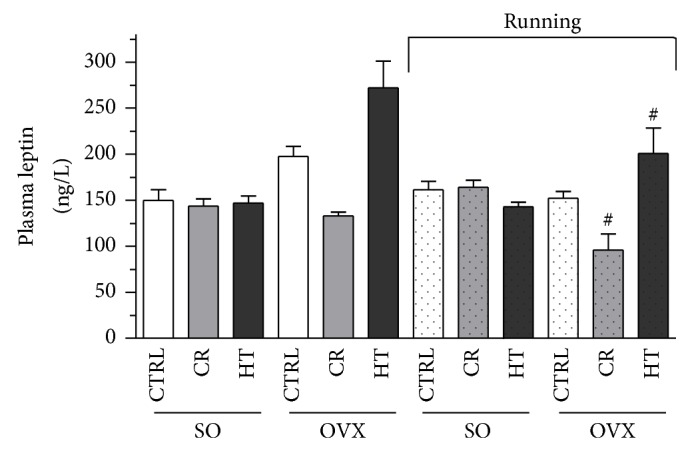
Effects of 12-week wheel-running exercise and nutrition on the plasma levels of leptin (expressed in ng/L). Means ± S.E.M. *n* = 10. Statistical significance: ^*^ *P* < 0.05 relative to the SO CTRL group, and ^#^ *P* < 0.05 is the significant difference between the running (R) and nonrunning groups. SO = sham-operated, OVX = ovariectomized, CTRL = standard chow, CR = caloric restriction, and HT = high-triglyceride.

The plasma leptin levels were measured by ELISA in each group. As compared with the SO animals, estrogen deprivation caused a significant increase in leptin level. The CR combined with 12 weeks of exercise decreased the leptin values similarly in both the SO and the OVX rats (28%, *P* < 0.05). The exercise training was effective only in the OVX HT subgroup (26%, *P* < 0.05). Data are presented in Figure 4.

### 3.6. Plasma AST and ALT Levels

**Figure 5:**
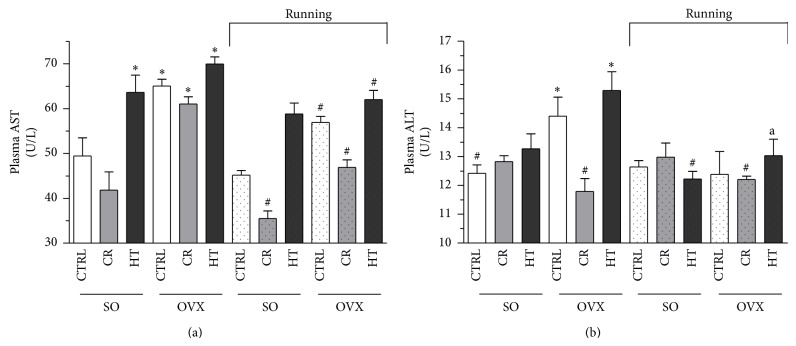
(a): Effects of 12-week wheel-running exercise and nutrition on the plasma levels of AST (expressed in U/L). Values are means ± S.E.M. Statistical significance: ^*^ *P* < 0.05 relative to the SO CTRL group, and ^#^ *P* < 0.05 is the significant difference between the running (R) and nonrunning groups. SO = sham-operated, OVX = ovariectomized, CTRL = standard chow, CR = calorie restriction, HT = high-triglyceride. (b): Effects of 12-week wheel-running exercise and nutrition on the plasma levels of ALT (expressed in U/L). Means ± S.E.M. Statistical significance: ^*^ *P* < 0.05 relative to the SO CTRL group, and ^#^ *P* < 0.05 is the significant difference between the running (R) and nonrunning groups. SO = sham-operated, OVX = ovariectomized, CTRL = standard chow, CR = calorie restriction, and HT = high-triglyceride.

The concentrations of AST and ALT showed that both were augmented in the OVX group HT diet which caused increases in the enzyme levels (AST: 41%, *P* < 0.05; ALT: 23%, *P* < 0.05). It emerged that 12 weeks of voluntary exercise or CR were effective, but the most marked reduction was observed from the combination of exercise and CR. Data are shown in Figure 5.

## 4. Discussion

Epidemiological, clinical, and molecular studies have shown that estrogen and estrogen receptors play an important role in metabolic homeostasis [7] through influencing fat metabolism, regulating the activity of molecules involved in orexigenic action, and regulating the neuronal activity of energy homeostasis [8], and the loss of estrogen may have profound effects on the glucose homeostasis and the body composition both in menopause women [9] and in rodents [4, 10].

The estrogen deficiency after menopause leads to becoming overweight or obesity, and physical exercise is one of the important modulators of this body weight gain [11]. The chronic consumption of a HT diet in rats induced MS, as evidenced by visceral obesity, hyperglycemia, dyslipidemia, an endothelial dysfunction, and hypertension [12]. In the present study, we set out to investigate the combined effects of physical activity and nutrition on estrogen-depleted OVX rats. We observed that the combination of the dietary control with exercise training was more effective in reducing the body weight, improving the leptin regulatory processes, and restoring the insulin, glucose, triglyceride, AST, and ALT levels in the plasma.

It is widely accepted that the prevalent lifestyle model of western societies, characterized by limited physical activity, an excessive calorie intake, and repetitive behavioral patterns contributes to dysregulation of the otherwise homeostatic control of body weight. In addition to physical activity, the reduction in energy intake may play a key factor in the modulation of life span. Extension of maximal life span is currently possible in animal models with CR. CR appears to prolong life by reducing reactive oxygen species (ROS)-mediated oxidative damage. Cornelius et al. discuss the role of CR on longevity processes by activating vitagenes which are involved in preserving cellular homeostasis during stressful conditions. They illustrate a complex network in which CR and hormetic CR-mimetics compounds by activating vitagenes can enhance defensive systems involved in bioenergetics and stress resistance homeostasis. Beside the anatomical and Mendelian paradigms, this approach may help to facilitate healthy lifestyle [13, 14].

Our experimental findings agree with those from other investigations in that the combination of exercise and diet modification has good effects on the metabolic parameters [15, 16]. As the augmented metabolic parameters correlated positively with cardiovascular disease-induced metabolic disorders [15, 17–21], our experimental findings are strongly supportive of the benefit of exercise as a means of reducing the metabolic parameters and hence the cardiovascular risk.

An improved insulin sensitivity is a hallmark outcome of exercise training: importantly, endurance training can restore the insulin response in obese, insulin-resistant rodents, and humans [22, 23]. Insulin resistance is a common condition in obesity, type 2 diabetes, dyslipidemia, and hypertension and there is experimental evidence that insulin resistance and hyperinsulinemia precede the development of obesity and other MS factors [24]. Exercise training may be one of the preventive and therapeutic strategies against impaired leptin and insulin signal transduction in the hypothalamus of obese individuals and associated with the markedly increased phosphorylation or activity of various proteins involved in leptin and insulin signal transduction [25, 26]. Riant et al. observed impaired glucose tolerance and insulin resistance in OVX mice fed with high fat diet [27]. We have shown that rats fed with a HT diet display hyperinsulinemia and hyperglycemia, which can be improved by physical exercise combined with dietary restriction.

The HT diet seems to influence the glucose metabolism in the exposed animals. The serum levels of insulin underwent significant changes in the OVX rats as compared with the SO rats, which could be taken as an indication that they were progressively developing an insulin-resistant condition.

It is well established that an increase in plasma triglyceride level elevates the risk of the development of atherosclerosis and dramatic clinical events such as acute myocardial infarction and stroke. Furthermore, lowering the plasma triglyceride content can reverse the progression of atherosclerosis and prevent cardiovascular events [28]. As an essential component of the metabolic syndrome, the blood triglyceride level was also investigated in this study. Plasma triglyceride levels were significantly higher in ovariectomized rats fed with HT but these high values were suppressed by exercise training. The exercise training associated with CR, especially, was the most effective therapy in OVX rats. The beneficial effects of estrogens on the blood lipid profile have long been known [29]. Physical activity is likewise well known to act on the blood lipid profiles in a positive manner [30]. Our study revealed significant effects of their combination and we believe that the combination may similarly result in additive effects with respect to the prevention of arteriosclerosis. We earlier demonstrated significant differences in hemeoxygenase and nitrogen monoxide synthase activities and cardiovascular parameters between male and female rats and revealed the anti-inflammatory effects of voluntary exercise training; in this respect, related investigations on the cardiovascular tissue of these animals are ongoing [31, 32].

Leptin hormone, secreted in the periphery by fat cells, plays an important role in the endocrine system; it signals the status of the body's energy stores and downregulates feeding behavior, regulating the appetite and energy expenditure [33]. Leptin is required for energy stores to be sensed in the central nervous system and is therefore essential for functions such as the normal energy homeostasis and reproduction [34]. Leptin is an adipocytokine that is mainly expressed in adipose tissue [34]. It is able to resist insulin secretion and exhibits a positive correlation with the body fat content [35].

Kang et al. reported that the plasma leptin level was significantly increased in a high-fat diet group as compared to a high-fat diet combined with training group and suggested that the effect of leptin sensitivity in the periphery may primarily relate to combined dietary control and exercise training more than to dietary control alone [16]. Short-term and long-term calorie restriction without exercise training has been shown to reduce plasma leptin levels dramatically in obese humans and rats [36–38]. Components other than physical exercise can play a role in the regulation of feeding behavior and the related mechanisms in developing mice [39]. The reduction in fat deposition with training in OVX rats raises the question of whether the level of leptin is improved in these rats.

Nonalcoholic fatty liver disease (NAFLD) is emerging as an acknowledged component of the MS. Markers of this condition, such as elevations in the serum concentrations of AST and ALT, may be considered reliable predictors of the development of the MS [40]. OVX resulted in higher body weights and augmented the AST and ALT levels as compared with those of the SO rats. Cameron et al. demonstrated that endurance exercise normalized the plasma AST and ALT levels in a diet-induced MS rat model [12]. Other studies have revealed the presence of NAFLD in rats that consumed a high-fat diet (71% of the energy intake from fat) for 3 weeks. Similarly, our data indicated high plasma levels of ALT and AST [41, 42]. Plasma AST and ALT levels were significantly higher in ovariectomized rats fed with HT but these high values were suppressed by exercise training. We found that both 12 weeks of exercise or CR alone was effective, but the most marked reduction was observed from the combination of exercise and CR.

After a 12-week HT diet, the OVX rats in the present study exhibited some symptoms of the MS. OVX nonrunning rats exhibited a significantly higher final body weight, and augmented AST, ALT, triglyceride, and leptin levels. These effects were prevented, in part, by exercise or a CR, but most effectively by a combination of the two. This indicates that the prevention of a weight gain may be a result not only of exercise training but also of metabolic changes under the control of estradiol. This indicates that exercise training has a strong influence in lowering the body fat accumulation following a decrease in estrogen levels.

In summary, the results of the present short-term investigations indicate that combined therapy involving a CR and exercise training can exert a positive influence on parameters related to the lipid metabolism in OVX rats. It is our conclusion that our data provide evidence that a CR combined with physical activity can be a very effective strategy for prevention of the development of an MS induced by high calorie diet.

## Figures and Tables

**Table 1:** Body weights at the start and end of the training and feeding period.

Subgroup	Body weight (g) at the start of the training and feeding period (0 w)	Body weight (g) at the end of the training and feeding period (12 w)
SO CTRL	279 ± 5.5	354 ± 9.2
SO CTRL R	273 ± 9.9	331 ± 9.0
SO CR	283 ± 7.6	263 ± 5.3^*^
SO CR R	261 ± 6.1^∗#^	234 ± 12.4^∗#^
SO HT	282 ± 11.0	319 ± 11.3^*^
SO HT R	269 ± 6.1	325 ± 7.8^*^
OVX CTRL	300 ± 7.2^*^	349 ± 21.9
OVX CTRL R	321 ± 9.4^*^	378 ± 14.2
OVX CR	334 ± 10.1^*^	305 ± 9.3^*^
OVX CR R	312 ± 9.3^*^	253 ± 25.7^*^
OVX HT	309 ± 6.7^*^	377 ± 12.0
OVX HT R	326 ± 7.5^*^	393 ± 13.7^*^

Results are shown as means ± S.E.M.

Statistical significance: ^*^ *P* < 0.05 relative to the SO CTRL group at the start and end of the training and feeding period, and ^#^ *P* < 0.05 is a significant difference between the running (R) and nonrunning groups.

SO: sham-operated, OVX: ovariectomized, CTRL: standard chow, CR: calorie restriction, HT: high-triglyceride, and R: running.
